# Comparing episodic memory outcomes from walking augmented reality and stationary virtual reality encoding experiences

**DOI:** 10.1038/s41598-024-57668-w

**Published:** 2024-03-30

**Authors:** Alvaro Pastor, Pierre Bourdin-Kreitz

**Affiliations:** 1 XR-Lab, Research-HUB, Universitat Oberta de Catalunya, Barcelona, Spain; 2 Computer Science, Multimedia and Telecommunication Department, Universitat Oberta de Catalunya, Barcelona, Spain

**Keywords:** Episodic memory, Virtual reality, Augmented reality, Locomotion, Spatial navigation, Face recognition, Architectural features, Human behaviour, Computer science, Long-term memory

## Abstract

Episodic Memory (EM) is the neurocognitive capacity to consciously recollect personally experienced events in specific spatio-temporal contexts. Although the relevance of spatial and temporal information is widely acknowledged in the EM literature, it remains unclear whether and how EM performance and organisation is modulated by self-motion, and by motor- and visually- salient environmental features (EFs) of the encoding environment. This study examines whether and how EM is modulated by locomotion and the EFs encountered in a controlled lifelike learning route within a large-scale building. Twenty-eight healthy participants took part in a museum-tour encoding task implemented in walking Augmented Reality (AR) and stationary Virtual Reality (VR) conditions. EM performance and organisation were assessed immediately and 48-hours after trials using a Remember/Familiar recognition paradigm. Results showed a significant positive modulation effect of locomotion on distinctive EM aspects. Findings highlighted a significant performance enhancement effect of stairway-adjacent locations compared to dead-end and mid-route stimuli-presentation locations. The results of this study may serve as design criteria to facilitate neurocognitive rehabilitative interventions of EM. The underlying technological framework developed for this study represents a novel and ecologically sound method for evaluating EM processes in lifelike situations, allowing researchers a naturalistic perspective into the complex nature of EM.

## Introduction

### Episodic memory

Episodic Memory (EM) is the neurocognitive capacity to consciously recollect personally experienced events in their spatio-temporal context^1^. EM maintains an updated representation of events and their context by associating multimodal information about “What”, “When” and “Where” in coherent episodes through a process known as Binding^2–4^. As in a mental time-travel, the phenomenological experience of remembering an event reinstates that a particular event occurred at a particular time, in a particular place, together as a coherent experience.

In this view, an event has been defined as a temporally localized change in the perceived state of the world^5^. The integrated memory for an event, a time, and a place (i.e., Binding of “What”, “When”, “Where”) constitutes the basic hallmark of EM^6^. Approaching this integrated memory content by dividing it into item (i.e., the contents of an event), and source (i.e., the context in which the item was experienced) has proven to be influential in EM research^7^. Indeed, it has been shown that different brain processes make distinct contributions to item and source memory contents^8,9^.

Early evidence showing discrete brain structures engaging with different aspects of EM came from the patient H.M. who suffered anterograde amnesia after bilateral resection of medial temporal lobes for treatment of epilepsy^10,11^. The hippocampus has since been linked to spatial navigation, with specific neural structures exhibiting location specific activation^12^. These discoveries gave way to the *Cognitive Map* theory which regards the hippocampus as the relational constructor of allocentric spatial maps of the environment^13,14^. This role of relational constructor has been proposed as the link between spatial navigation and EM, considering that one of the key defining properties of EM is spatio-temporal context^15,16^.

The importance of context in EM has been more recently formalized into theories proposing that spatial context serves as a scaffold for the relational construction of EM^17,18^. This has been extended to a role of the hippocampus in the relational construction of coherent spatial scenes during imagination processes^19,20^. Furthermore, a growing body of evidence from functional imaging and neuropsychological studies has indicated that the hippocampus is a key neural structure involved in the long-term Binding processes that compose discrete coherent episodes out of representations of items and sources^21–23^. Studies have shown that the hippocampus is active during encoding of multiple items and especially during systematic comparisons between items and not during rote rehearsal of individual items^24^. Past research has revealed activation within hippocampus during encoding of face-name associations^25,26^. The neural systems that support EM would implicitly and continuously construct relational models, not only to organize and process immediately experienced events, but extrapolating beyond the boundaries of the current field of view in order to make predictions of the upcoming environment^18,27,28^.

Importantly, the continuous context representation of the EM system has been found to be adapted at the encounter of physical spatial boundaries^29,30^, as part of an automatic process triggered by detecting perceptual changes^31–33^. This would strongly suggest that environmental features (EF) may influence EM performance and the organisation of memory episodes^34,35^. Studies have shown a role of physical spatial boundaries in weakening the links between preceding and subsequent information, organizing experience into discrete episodes^5^. Specifically, it has been observed that doorways serve as event boundaries, and have a direct impact on recollection over a retention period of two weeks^36–39^. In contrast to doorways, less research has been conducted on other EFs which could provoke the segmentation of experience into episodes^40–42^.

In this regard, in the view of *Event Segmentation Theory*, episodic memories are segmented by shifts in context that engage prediction processes of near-future experience known as *event boundaries*^43,44^. Past research has also shown that items encoded in spatially proximal locations tend to maintain cluster in recall, a phenomenon known as spatial clustering^45^. During EM encoding processes, contents of experience would become automatically associated with spatial and temporal context. During retrieval, prior context would become partially reinstated, helping to cue other events experienced within the same or related context^46^. According to context-retrieval models^47,48^ episodic retrieval would be organised in clusters of items that share associated context, and these clusters would reflect the abrupt context shifts experienced at encoding^49^.

To understand how the environment imposes sensorimotor adaptations and influences brain dynamics independent of awareness, past research has established a distinction between EFs and sensorimotor responses (SMR)^50–56^. Specifically, the current study considers the coupling between EF and associated SMR that could have an impact on EM outcomes^57–59^. Among theories of the dynamic interrelations between EF and SMR in embodied cognition, this study followed the empirically grounded theory of *sensorimotor contingencies* (SMC)^60,61^. SMC focuses on the interactions between organism and environment so that motor actions and associated sensory consequences are linked together by adaptive relations or SMCs^62–66^. Emphasizing the critical role of action, SMC posits that perception arises from an intrinsic knowledge of how actions within an environment would alter sensations, leading to a specific perception. Additionally, SMC explains behavior by framing it in the context of inference regarding the origins of proprioceptive sensations. This explanation is built on a model of active inference, in which higher cortical levels send descending proprioceptive predictions, rather than motor commands. This process mirrors perceptual inference in the sensory cortex, where descending connections convey predictions, while ascending connections convey prediction errors^67–69^. Hence, a distinctive pattern of SMCs in a given environment may provide behavioural conditions and opportunities with predictable outcomes, while a change in SMCs would demand an update of estimates of the posterior outcomes^70,71^. In this regard, SMC seems to provide further support for the effects of event boundaries on episodic segmentation and *Event Segmentation Theory*^31–33,43,45,49,72^.

### Recognition

Recognition in EM studies has been described as a composite of two complementary processes: recollection and familiarity^73^. Recollection, i.e., remembering, indicates a subject retrieving both item and contextual information together with a strong impression of when and where an event took place^74,75^. In contrast, the feeling of familiarity would be caused by integration of item-specific information without reaching the contextual information threshold needed for recollection i.e., remembering^7^. Recollection involves remembering the stimulus in the context associated with the experience, and familiarity involves a sense of having encountered the features of the stimulus before, but without any specific details about the context^76,77^.

Recollection and familiarity have been shown to be functionally dissociable and to rely on partially separable brain regions^77,78^. In addition, recollection and familiarity have been associated with distinct event related potential modulations^79,80^. Although the distinction between recollection and familiarity is relatively well established, issues remain about how to best separate these processes in cognitive tasks^81^.

The availability of context-rich knowledge has been proposed as the key characteristic for recollection but not familiarity^82^. In this regard, one approach is to use tests of source memory as measures of recollection (i.e., tasks that require subjects to retrieve context of a studied item), and compare this to performance on tests of item recognition (i.e., tasks that require subjects to discriminate between studied and new items). The idea is that, if accurate source discriminations rely exclusively on recollection then performance on these tests can be used as an index of recollection. In contrast, if an item is recognized as remembered but leads to an incorrect source memory judgement then this can be interpreted as a process distinct from recollection^83^.

An alternative approach is to estimate recollection on the basis of recognition confidence judgments. The idea is that the recollection of qualitative information about a study event should lead to confident recognition memory responses, whereas familiarity in the absence of recollection should support lower confidence recognition responses. Thus, in standard item recognition tests recollection should be restricted primarily to high confidence recognition responses whereas familiarity should increase gradually across levels of confidence^84^. Correct source judgements are usually associated with the highest level of recognition confidence, so these two approaches often lead to the same conclusions. In instances where subjects can recognise items with low confidence and subsequently accurately discern the source of those items, past research has indicated that source recognition for low-confidence item hits is supported by a neurocognitive process distinct from recollection.^83^.

Recent studies have shown that the hippocampus instead of being exclusively engaged in one of these processes, contributes to both recollection and familiarity in dynamic interactions depending on task and availability of neural resources^85,86^. Importantly, familiarity is not expected to support associative memory for two distinct items (e.g., between-item Binding), unless the two items can be treated as a single larger item as part of a within-item Binding processing (i.e., as a mouth, nose, and eyes can form a single entity known as face)^7^.

#### VR and AR tools

As previous research has shown, the spatio-temporal aspects of experiences are key components of EM encoding and recall. However, a great part of traditional neuropsychological EM tests have been criticised for their lack in ecological validity i.e., the degree in which experimental settings are similar to real-world conditions^87–89^. In this regard, a large part of traditional approaches have been criticised for shortcomings in the prediction of episodic performance beyond laboratory conditions.

Over the past decade, virtual reality (VR) and augmented reality (AR) technologies have gained considerable interest as platforms for creating controlled yet ecologically valid experimental environments. These technologies offer the capability to simulate lifelike events, making them applicable across various scientific domains, including episodic memory (EM) research.^90,91^. Participants in a VR scenario can behave as if they were in the real world^92^. Three main features of VR may have implications for EM research and rehabilitation.

First, VR and AR environments can be designed to offer high ecological validity, referring to the degree to which a test is similar to real-world task demands and user performance. This has facilitated new insights into the activity of brain regions involved in tasks that were difficult to address in a naturalistic manner using traditional neuropsychological tests. For instance, spatial cognition and navigation, motor tasks, multisensory integration, and social interaction^93–96^. Another important potential is that VR can provide realistic, feature-rich environments that can be implemented with different degrees of complexity. Past studies have demonstrated that complex virtual environments can stimulate hippocampal activity which in turn improves EM^97^.

Second, VR can allow EM researchers to manipulate the participant’s point of view on the actions taking place in a virtual environment. In this way, VR could offer a unique avenue for studies of body representation and ownership and its influence on a variety of cognitive processes^98^. In line with this, past studies^99,100^ that examined point of view in relation to encoding and retrieving real-life events, revealed a poorer recollection for life events encoded in an out-of-body frame of reference, compared to participants in an in-body subjective frame of reference.

Third, VR can allow researchers to manipulate navigation techniques and degrees of involvement of participants in an encoding task. This in turn could help examine active and passive navigation effects on EM. Results from past studies have suggested that active navigation can benefit EM processes^101–103^.

The inherently situated nature of EM, exemplified in everyday tasks such as navigating an environment without getting lost or retrieving memories of personal experiences, require the continuous processing and encoding of spatial information concerning the location of an individual, movements, and the EFs surrounding experience^104,105^. As deficits in navigational and EM functions are a hallmark symptom of memory-related disorders such as Alzheimer’s Disease (AD) and amnestic mild cognitive impairment (aMCI)^106,107^, spatial information is of critical importance for research on EM and development of therapies^108^. Considering the key spatio-temporal aspects of EM processes, VR and AR can help situate experimental designs in real-world contexts or their virtual representations, including common distractors and interferences. The tasks ’premises and the actions performed by subjects in VR and AR can be designed to closely relate to real-world conditions, and to address specific processes relevant for naturalistic observation of EM.

Prior studies have used feature-rich realistic environments, in a range from large-scale places such as a part of a town or a shopping mall walk-through^109–111^, or room-scale locations such as a home, an office, or a grocery shop^103,112,113^. In contrast to VR, the number of AR systems developed specifically to study EM processes is much smaller^114^. Nevertheless, previous studies comparing image recognition, spatial navigation and visual processing outcomes from AR and VR environments have highlighted that AR and VR can be comparable depending on the specific setup and study objective^115^. For instance, it has been shown that different manipulations of field of view and realism of lighting in an item/source recognition task had no significant effect on recognition outcomes^116^. In object recognition tasks comparing devices including handheld AR and HMD-based VR, past research has shown no significant differences in immediate object recognition^117^. Using a mobile electroencephalogram system and brain signals from the face inversion effect as relevant measures, a recent study found no significant differences between a lab-based computer task, walking through an indoor environment while seeing face photographs, and walking through an indoor environment using handheld AR^118^.

Finally, some VR and AR methods have been validated in relation to traditional neuropsychological assessment methods. For instance, results from the “Virtual Environment Grocery Store”^119^ and the California Verbal Learning Test were correlated. Also, the “Virtual Reality Everyday Assessment Lab”^120^ and the “Virtual Shop” showed validity to examine and train episodic memory in older adults^121,122^. In the field of spatial memory and navigation, “VRmaze” is an example that implements three of the most widely used experimental mazes, maintaining correlation with the results obtained by traditional methods^123^. Similarly, AR tools have shown validity in the assessment of specific cognitive aspects. In the assessment of spatial memory AR frameworks has shown significant correlations to outcomes obtained via traditional approaches^124–126^.

#### The current study

This study approaches EM using AR and VR in a stimuli-rich real-world environment, to examine whether and how EM is modulated by locomotion and the EFs encountered in a lifelike learning experience. To this end, the present experimental study was conducted in a public Museum, using a *museum-tour* encoding task implemented with two conditions: walking AR and stationary VR.

To carry out this investigation, the employed recruitment strategy prioritised gathering a wide range of ages within the healthy adult population. The aim was to satisfy the criteria of past studies in which the adult-elderly threshold relevant for EM was set approximately at age 60^127–132^. This criterion was based on the key importance of age in EM^133–137^, age-related decline in spatial navigation^138–140^, decline in precision in location, color and orientation memory for objects^141^, differential neural recruitment in encoding tasks^142,143^ and importantly, decline in recognition memory for pictures^144^, memory associations^145–148^ and item and context memory^149^.

This study pursued two objectives. First, to assess the effect of locomotion on EM, comparing EM outcomes from participants in walking AR and stationary VR conditions. While previous studies have examined the effect of spatial exploration in EM using VR in laboratory settings^45,101^, few experiments have compared EM outcomes between walking and stationary encoding experiences in large-scale settings^150–152^.

Second, to examine whether and how stairways influence EM, comparing performance and organisation measures. In this study, a characterization of the available stimulus-presentation locations was conducted using an approach following Djebbara et al.^153^, exploring how the EF of the built environment may contribute to the neurodynamics of EM^59,154–156^. Adhering to the SMC perspective, the current study employed a sequential approach, from least to most demands of change on SMC to categorise the available experimental stimulus-presentation locations into three distinct classes. The assessment of SMC change was based on pre- and post-stimulus presentation, with the start of encoding for each stimulus serving as the central event in this process^157^. The stimulus-presentation locations used in this study encompassed three classes: dead-end locations (i.e., least change), mid-route locations (i.e., moderate change) and stairway-adjacent locations (i.e., most change).

Two hypotheses have been formulated considering these objectives:

- H1: Do walking-enabled encoding experiences affect EM compared to stationary encoding in “What”, “Where”, “When” or Binding?
  Considering that previous studies have demonstrated a beneficial effect of exercise during early memory consolidation^158–161^, and that walking may benefit the ability to build an accurate cognitive map of the experience^162,163^, it was expected that participants of the walking AR condition would have more probability to build an accurate cognitive representation of the environment, together with an accurate localisation of the encoded target items. Specifically, it was expected that locomotion would positively modulate the measures of “Where”, “When” and Binding.
- H2: Do environmental features (EFs) influence EM performance and organisation?
  Considering that prior research has shown an EM modulation effect through the influence of route turns^30^ and doorways^151,164–166^, it was expected that stimulus-presentation locations with distinct EF and SMC would result in distinct EM performance and organisation outcomes. The expected effect was twofold. First, locations that induced more substantial changes in visuomotor engagement with the environment between pre- and post-stimulus presentation were expected to yield better EM outcomes compared to less demanding stimulus-presentation locations^31,43,72^. Second, EFs producing a sudden shift in SMC, particularly in visuomotor engagement with the environment, were expected to yield an event boundary effect, segmenting the encoding experience into distinct clusters. Consequently, an enhancement in EM accuracy was expected for items within the same cluster compared to those across different clusters. Distinctive variations were expected in relational measures between pre- and post-stairway encoding events, including order judgments and associative inferences between faces and places^45,49^.

In the subsequent section, we will present the results obtained, followed by the discussion. Additionally, we have included a detailed Methods section outlining participant information, materials, measures, procedure, and finally a section on statistical analyses employed in this study.

## Results

This study pursued two objectives. First, to assess the effect of locomotion on EM, comparing EM outcomes from participants in walking AR and stationary VR conditions. Second, to examine whether and how stairways influence EM, comparing performance and organisation measures between stairway-adjacent, mid-route and dead-end stimuli-presentation locations. To achieve these objectives, a lifelike museum-tour encoding task was conducted in AR and VR experimental conditions to compare EM outcomes “What”, “Where”, “When” and Binding via Immediate Questionnaire (IQ) and Delayed Questionnaire (DQ). Twenty-eight participants completed the IQ, and twenty-five the DQ.

### Preliminary

As described in the Methods section, participants from a wide a range of ages were recruited among the healthy adult population in Barcelona, and distributed between the two VR and AR experimental encoding conditions using age-based stratified sample randomisation procedures. A post hoc Levene test for equality of variances confirmed the **homogeneity of participants’ ages between conditions** $(F(1,26) = 0.17,\ p =.69)$. Confirming the even distribution of ages between conditions, a Mann-Whitney U test was performed considering the age of the participants, which did not show significant differences between conditions $(U=95.500 ,\ p=.93)$.

A uniform encoding route was implemented for AR and VR conditions. Three participants in the AR condition skipped up to three items in the encoding tour out of a total of 13 available presentation-events stimulus. Hence the number of stimuli encountered by the AR group varied in a range from 10 to 13 $(Mdn=13 ,\ M=12.6 ,\ SD=0.6)$. The possible impact of these missing items on the measurements was assessed through different analyses. A repeated measures analysis of variance (RMANOVA) was carried out to compare the variances in the number of questionnaire items available to each subject between conditions, using the seventeen different sections of the IQ and DQ as levels in the analysis. This RMANOVA revealed no significant differences between conditions $(F(1,26)=3.01 , p = .09 ,\ \omega ^2\ =0.04)$. The duration of the VR encoding tour was predetermined at 818 seconds, while in the AR condition, the duration varied due to participants’ self-defined walking pace, recorded within a range of 599 to 938 seconds $(n=14 ,\ Mdn=805.0 ,\ M=784.3 ,\ SD=104.9 )$. A Kruskal-Wallis Test demonstrated no significant differences $(H(1) =1.89,\ p=.17)$ in tour duration between conditions. Another RMANOVA was carried out to compare the variances in interstimulus intervals (ISI) between AR and VR conditions, considering ISI in seconds as measures, showing no significant differences $(F(1,23)=2.32 ,\ p=.15 ,\ \omega ^2\ =0.02)$. **These findings underscored the comparability of the encoding experiences in VR and AR conditions**.

Additionally, in the analysis of participants’ responses regarding **familiarity with the Museum building** (i.e., the experimental site where the museum-encoding tour was implemented), the data revealed a low level of familiarity with the Museum building among participants in both the AR condition $(n=14,\ M= 0.41,\ SD=0.22)$ and the VR condition $(n=14,\ M= 0.28,\ SD=0.25)$. To confirm the comparability of this prior knowledge of the experimental site between conditions, a Mann-Whitney U Test was executed using scores derived from the linear transformation of 5-point Likert scale responses, indicating no significant difference between conditions $(n=14 ,\ U=131.00 ,\ p=.13 ,\ r=0.19)$.

Finally, a self-report Discrete Emotion Questionnaire (DEQ) was collected at the beginning of the the IQ and the DQ, to ensure that no participant suffered from anxious, stressed or depressed emotional states that could hinder their retrieval performance. The emotions most frequently reported by participants at the beginning of IQ were **Happiness** (26/28, 93%) and **Surprise**, (24/28, 86%). **Happiness** scores for the AR $(n=14,\ Mdn=0.5,\ M=0.4,\ SD= 0.3)$ and VR conditions $(n=14 ,\ Mdn=0.5 ,\ M=0.4 ,\ SD=0.2)$ were used in an ANOVA test, showing no significant difference between conditions. Ratings of **Surprise** from the VR condition $(n=14 ,\ Mdn=0.6 ,\ M=0.6 ,\ SD=0.2)$ and the AR condition $(n=14 ,\ Mdn=0.3 ,\ M=0.3 ,\ SD=0.3)$ showed a significant difference in ANOVA $(F(1,26)=5.92 ,\ p =.03 ,\ \omega ^2\ =0.15)$. Post-hoc testing using Bonferroni correction showed that AR participants were significantly less surprised than their VR counterparts at the beginning of the IQ $(Pbonf=.02,\ d=-0.92)$. The self-report DEQ at the beginning of the DQ, showed no report of anxious, stressed or depressed participants. The most commonly reported emotion was **Happiness** (20/25, 80%), followed by **Surprise** (7/25, 28%). An ANOVA test showed no significant differences between conditions for **Surprise** or **Happiness** self-reports at the beginning of DQ.

### “What”, “Where”, “When” aspects of EM

**Figure 1:**
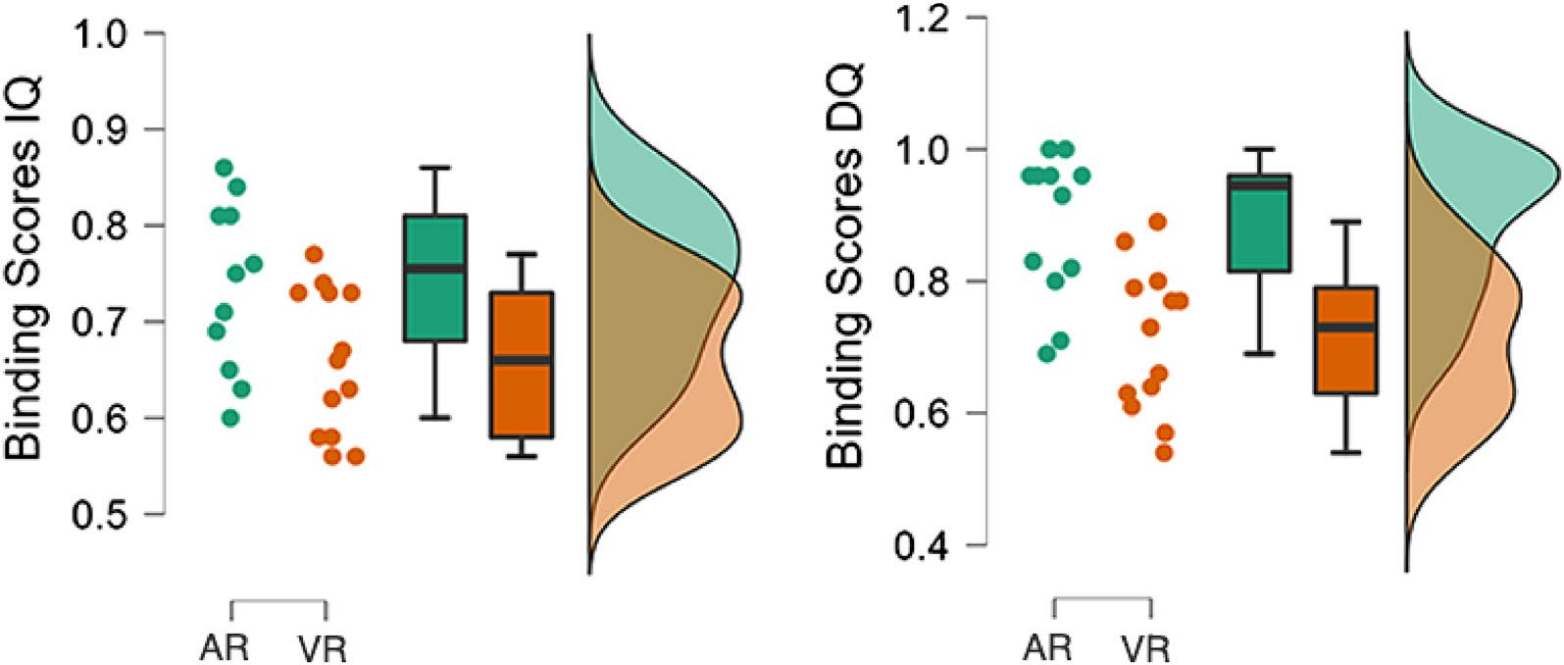
Results of RMANOVA on Binding scores using data from IQ (Left) and DQ (Right) measures showed a significant advantage $(p<.001)$ of AR condition. A post-hoc comparison between conditions using Bonferroni correction showed a large-size effect in favour of participants in the AR condition $(Pbonf<.001,\ d=1.31)$.

- **What**: A Mann-Whitney U Test was carried out using the accurate responses from **“What”** section of IQ, and revealed no significant differences between experimental conditions $(n=14,\ U=117.00,\ p=.19,\ r=0.19)$. Likewise, using accurate responses from DQ measures, a Mann-Whitney U Test showed no significant differences between conditions $(n=12,\ U=95.50,\ p=.17,\ r=0.22)$.
- **Where**: Next, comparing “Where” IQ accurate responses between conditions, a Mann-Whitney U Test showed no significant differences $(U= 122.00 ,\ p=.14 ,\ r=0.24)$. Some differences in favour of the AR condition were observed on the accuracy of **“Where”** responses at DQ $(U= 103.50,\ p=.06,\ r=0.33)$, although with no statistical significance.
- **When**: The first measure that examined the **“When”** aspect of EM, pertained to task duration estimation (only for the IQ), which showed no significant difference between the **estimated duration** and the actual tour duration between conditions $(U=120.000,\ p=.9,\ r=0.23)$. The second measure concerned a serial order task administered both at IQ and DQ. A Mann-Whitney U Test conducted on the IQ **face serial order** responses revealed a significant difference between conditions $(U=142.00,\ p=.02,\ r=0.45)$, indicating that participants in the AR condition had an advantage in correctly remembering the order of faces compared to participants in the VR group, with a medium-size effect. Similarly, a Mann-Whitney U Test applied on the DQ responses on **face serial order** revealed a similar significant effect $(n=12,\ U=112.50,\ p=.02, r=0.44)$ favouring AR condition participants, also with a medium-size effect.
  To examine responses from both IQ and DQ, a RMANOVA was performed on **face serial order** responses, showing a significant between-subjects effect of condition $(F(1,23)=7.08 ,\ p=.01 ,\ \omega ^2\ =0.11)$ with a medium-size effect. Using a post-hoc Bonferroni test, a significant difference was measured $(Pbonf=.01,\ d=0.93)$, with a large-size effect in favour of participants in the AR condition. A similar RMANOVA was performed focusing on **place serial order** responses, but found no significant differences between conditions $(F(1,22)=1.93 ,\ p=.18 ,\ \omega ^2\ =0.02)$. These results highlighted that participants in the AR condition were significantly more accurate in **face serial order responses** both at the IQ and DQ measures.
- **Binding**: The Binding aspect of EM was examined in this study via two questionnaire sections. The first addressed item-to-source (i.e., associated place) and source-to-item associations (i.e., associated face) seen together at encoding. The second focused on the examination of associative inferences between faces and places that were not seen together during encoding but shared a common spatial context.
  Considering associations of places and faces directly seen together at encoding, a Mann-Whitney U Test on the IQ responses showed no significant difference between conditions either in** associated place** $(U=101.0 ,\ p=.45 ,\ r=0.03)$ or **associated face** associations $(U=114.5,\ p=.23,\ r =0.17)$. However, examining DQ data with a Mann-Whitney U Test revealed a significant advantage of participants in the AR condition being more likely to correctly associate **faces to places**, with a medium-size effect $(n=12 ,\ U= 115.50 ,\ p=.01 ,\ r=0.47)$. Likewise, participants in the AR condition were significantly more likely to correctly associate **places to faces** than their VR counterparts, with a large-effect $(n=12,\ U= 126.50,\ p=.004,\ r=0.62)$. To examine responses from both IQ and DQ measures, a RMANOVA was carried out and showed a significant effect of condition on accuracy for **associated places** $(F(1,23)=5.58 ,\ p=.02 ,\ \omega ^2=0.09)$. In a post-hoc test using Bonferroni correction, an advantage in favour of AR condition participants was observed with a medium-size effect $(Pbonf=.02 ,\ d=0.79)$.
  The next set of analyses focused on associative inferences made from places and faces, and vice versa, not seen together at encoding. A Student’s T-test on the IQ responses of **face-based** associative inferences showed no significant difference between conditions $(t(26.00)=1.39,\ p=.094,\ r=0.51)$. While a Student’s T-test on the **place-based** associative inferences, showed a non-significant difference between conditions $(t(26.00)=1.49,\ p=.07,\ r=0.56)$. Regarding the DQ responses, a Mann-Whitney U Test revealed a significant difference with a medium-size effect in favour of AR condition participants on responses for **face-based** associative inferences $(U=121.00 ,\ p=.005 ,\ r=0.55)$.
  In order to examine responses from both IQ and DQ, a RMANOVA was carried out, focusing on associative inferences between conditions, showing a significant difference $(F(1,23)=12.13 ,\ p=.002 ,\ \omega ^2= 0.19)$ for **face-based** associative inferences with a large-size effect. A post-hoc comparison between conditions using Bonferroni correction showed a large-size effect in favour of participants in the AR condition $(Pbonf=.002,\ d=0.94)$. Similarly, a RMANOVA on IQ and DQ **place-based** associative inferences showed significant differences between conditions $(F(1,23)=4.55 ,\ p=.04 ,\ \omega ^2=0.07)$. A post-hoc comparison using Bonferroni correction pointed in favour of AR condition participants, with a medium-size effect $(Pbonf=.04 ,\ d=0.57)$.
  A Binding partial average was computed for IQ and DQ separately, considering the four measures described above (i.e., face-to-place, place-to-face direct associations, face-based and place-based associative inferences). A Mann-Whitney U Test showed a significant difference on the Binding average from IQ in favour of AR condition, with a medium-size effect $(U= 137.00 ,\ p=.04 ,\ r=0.40)$. A Mann-Whitney U Test carried out on the average DQ responses, and showed a significant difference in favour of AR condition, with a large-size effect $(U= 135.50 ,\ p<.001 ,\ r=0.74)$. These results highlighted that the **Binding aspect of EM was significantly more accurate in AR condition** both at the IQ and DQ responses. In order to further examine responses from both IQ and DQ, a RMANOVA was carried out, revealing a significant effect of condition on Binding aspect of EM (Figure 1) $(F(1,23)=18.27 ,\ p<.001 ,\ \omega ^2= 0.26)$. A post-hoc comparison between conditions using Bonferroni correction showed a large-size effect in favour of participants in the AR condition $(Pbonf<.001,\ d=1.31)$.

### Overall scores and comparisons

Using combined IQ and DQ responses, an overall average score was calculated for each EM aspect “What”, “Where”, “When” and Binding. A Mann-Whitney U Test carried out using **“What” overall average** did not show significant differences $(U= 125.00 ,\ p=.11 ,\ r=0.28)$ between conditions. Considering the **“When” overall average** a Mann-Whitney U Test did not show significant differences $(U= 125.00 ,\ p=.14 ,\ r=0.25)$ between conditions. A Mann-Whitney U Test using **“Where” overall average** did not show significant differences favouring AR condition participants $(U= 133.00 ,\ p=.05 ,\ r=0.36)$.

**Figure 2:**
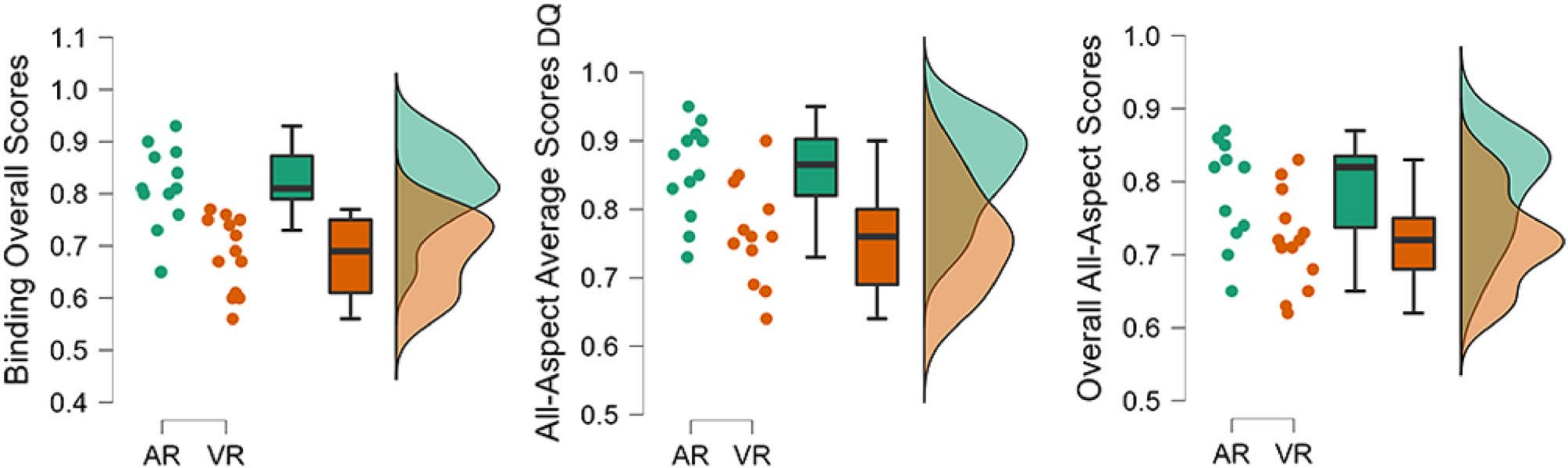
Left. Overall Binding scores showed significant benefit of AR condition with a large-size effect $(p=.003,\ r=0.61)$. Center: A significant advantage $(p=.003,\ r=0.64)$ of AR condition was observed on the overall average of the What, When, Where, Binding scores obtained from DQ data with a medium-size effect. Right: Comparisons of overall (i.e., across questionnaire-times) all-aspect EM scores between conditions showed a significant difference favouring AR participants with a small-size effect $(p=.04,\ r=0.38)$.

Following the same procedure, a Binding average combining IQ and DQ responses was calculated considering the item-to-source and source-to-item associations responses and face- and place-based associative inferences. A Mann-Whitney U Test on this **Binding overall average** showed significant differences in benefit of AR condition participants $(U= 157.50 ,\ p=.003 ,\ r=0.61)$ (Figure 2-Left), with a large-size effect. A Friedman test was conducted to determine differences between conditions in a RMANOVA, and the results showed significant differences $(x^2(1)=6.76, p=.009 ,\ W=0.27)$. A Conover Test was performed to clarify which condition had better Binding performance, revealing a significant advantage $(pBonf=.01)$ of AR condition.

To further characterise these results, an average from combined “What”, “Where”, “When” and Binding scores was calculated for IQ responses. On this average of immediate EM outcomes, a Mann-Whitney U Test indicated no difference between conditions $(U=130.00,\ p=.07,\ r=0.33)$. Similarly, an average delayed EM outcomes was calculated using **“What”, “Where”, “When” and Binding responses from DQ**. However, the Mann-Whitney U Test showed a significant difference in favour of AR condition participants (Figure 2-Center) $(U=128.00,\ p=.003,\ r=0.64)$, with a medium-size effect .

To finalise this characterisation, an overall score encompassing all aspects of episodic memory, termed the Overall Episodic Recall Score (OER), was computed by averaging “What”, “Where”, “When” and Binding responses from both IQ and DQ. A Mann-Whitney U Test indicated a significant difference favouring AR condition participants (Figure 2-Right) $(U=135.50 ,\ p=.04 ,\ r=0.38)$, with a small-size effect.

### Confidence self-reports from “When” and Binding responses

An average score was calculated from the confidence responses reported by participants in face serial order (“When”) and place serial order (“When”) sequences. Regarding assessment of participants’ responses at IQ, a Student’s t-test revealed a significant difference in responses, with large- and medium-sized effect of condition in confidence self-reports for **face order** $(t(26.00)=3.14,\ p=.002,\ d=1.18)$ and **place order** $(t(26.00)=1.70 ,\ p=.05 ,\ d=0.64)$. Considering the DQ responses, a Student’s T-test for **face order** responses, revealed a significant difference in favour of participants with a large-size effect $(t(23.00)=2.92,\ p=.004,\ d=1.17)$. A Mann-Whitney U Test was applied to **place order judgement confidence** reports from DQ, showing no significant difference between conditions $(U=105.00,\ p=.07,\ d=0.35)$.

Similarly, an average score was calculated using confidence reports from IQ and DQ correct responses of item-to-source (i.e., face to place Binding) and source-to-item (i.e., place to face Binding) associations. A Student’s T-test on IQ responses showed a significant difference in **item-to-source associations** with a large-size effect in favour of AR condition $(t(26.00)=2.13,\ p=.02,\ d=0.80)$. Regarding **source-to-item associations** a significant difference was found with a large-size effect in favour of AR participants, also using a Student’s T-test $(t(26.00)=2.76,\ p=.005,\ d=1.04)$. Considering DQ confidence reports a significant difference and large-effect sizes were found in favour of the AR condition, both in **item-to-source associations** $(t(23.00)=3.18 ,\ p=.002 ,\ d=1.27)$, and in **source-to-item associations** $(t(23.00)=2.72 ,\ p=.006 ,\ d=1.09)$.

To examine face-based and place-based associative inferences, an average score was calculated from confidence responses reported by participants from IQ and DQ separately. A Student’s T-test on IQ data showed a significant difference in favour of AR condition participants when performing **place-based associative inferences**, with a large-size effect$(t(26.00)=2.59 ,\ p=.008 ,\ d=0.98)$. Similarly, a significant difference was found in favour of AR condition participants when performing face-based associative inferences Student’s T-test performed on DQ responses $(t(23.00)=2.36 ,\ p=.01 ,\ d=0.95)$, with a large-effect size.

An association was examined between OER scores and an overall average of confidence scores per subject showing a significant positive correlation $(n=28 ,\ \tau b =0.27 ,\ p=.02)$.

### Influence of architectural features

To evaluate the potential impact of EFs on EM outcomes, this study categorized the available stimulus-presentation locations into three types. This classification followed a linear progression, ranging from locations which imposed the least to the most demands of change on participants’ SMC model of the environment.

**Figure 3:**
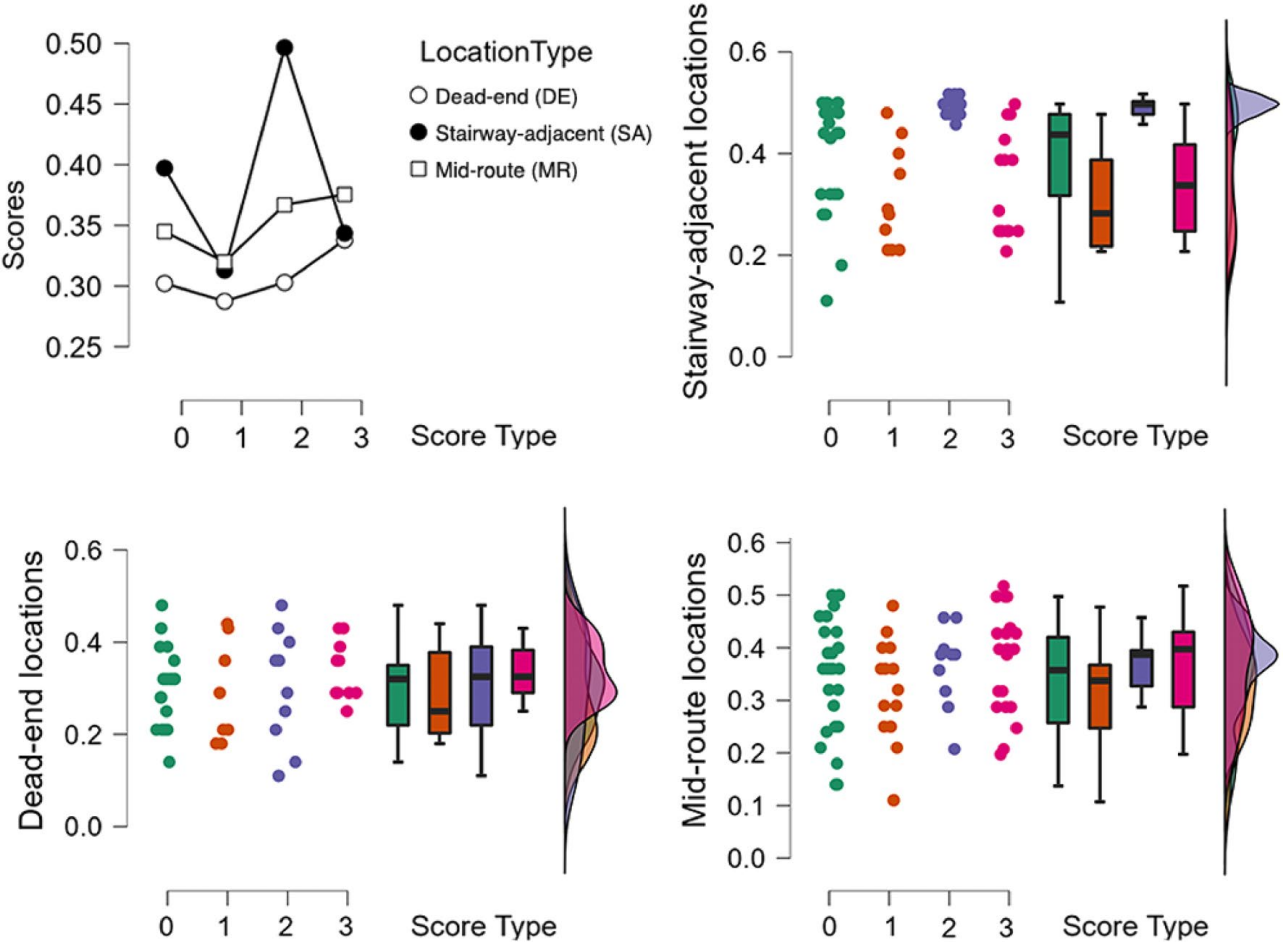
Four types of scores (i.e., direct item-source association (0), What (1), Where(2) and associative inferences (3)) were compared in the three location types available: dead-end (DE), stairway-adjacent (SA) and mid-route (MR). Top-Left. Descriptive overview of performances of the four score types at the three locations. Top-Right. Responses obtained at stairway-adjacent locations highlight the positive modulation effect of stairway on Where (2) responses and direct item-source associations (0). Bottom-Left. Scores obtained at dead-end locations. Bottom-Right. Responses obtained at mid-route locations.

Each subject’s accurate responses in face and place recognition, direct item-to-source and source-to-item association, and face- and place-based associative inference sections (i.e., the four types of scores : direct item-source association (0 in figure 3), What (1 in figure 3), Where (2 in figure 3) and associative inferences (3 in figure 3), compounded an **all-aspect average** for an ANOVA between location types. Results indicated a significant effect of location type on accurate responses $(F(2,170)=8.37,\ p<.001,\ \omega ^2=0.08)$, with a medium-size effect. A Kruskal-Wallis test was then performed and confirmed a significant difference between location types $(H(2) =16.78,\ p<.001)$. However, no significant interaction was found between conditions and location types $(F(2,170)=0.67 ,\ p=.51 ,\ \omega ^2=0.00)$. A Dunn’s test was performed to carry out pairwise comparisons, and revealed a significant advantage of **stairway-adjacent locations** compared both to dead-end locations $(p<.001 ,\ Pbonf < .001 ,\ z=-4.08)$, and mid-route encoding events $(p=.01,\ Pbonf=.04,\ z=2.27)$.

In the following analyses, accurate responses from face recognition, place recognition, item-to-source association, source-to-item association, as well as face- and place-based associative inferences were considered separately (Figure 3). A significant interaction was found between **location type and score type**, with a small-size effect $(F(6,164)=2.64 ,\ p=.018 ,\ \omega ^2=0.05)$. A significant effect of **location type** was also observed $(F(2,164)=7.98 ,\ p=<.001 ,\ \omega ^2=0.07)$ with a medium-size effect. Whereas the influence of **score type** was also significant $(F(3,164)=3.78,\ p=.012,\ \omega ^2=0.04)$ but with a small-size effect. Furthermore, a Kruskal-Wallis test confirmed the significant effects of location type $(H(2)=16.76,\ P<.001)$, and score type $(H(3)=11.97,\ p=.007)$. A Standard Post-Hoc test using Bonferroni correction was conducted to assess pairwise comparisons, and revealed that the **stairway-adjacent - “Where”** scores were significantly better in all comparative categories (4 score types in 3 location types) $(pBonf<.001)$, including when compared to dead-end - associative inferences $(pBonf=.012 ,\ d=1.64)$, and mid-route associative inferences $(pBonf =.006,\ d=1.58)$.

As the stairway element was observed as an EM-modulating architectural feature, the next analysis examined a clustering effect in order judgements for pre- and post-stairway stimuli expositions. To this end, face and place order responses were divided in pre-stairway and post-stairway stimuli presentation-events. A Mann-Whitney U Test revealed a significant advantage $(U=68.00,\ p=.046,\ d=0.54)$ of pre-stairway **face targets** being recalled in correct order irrespective of condition, in comparison to those encountered post-stairway.

## Discussion

This study reports an experiment that compares EM outcomes resulting from a realistic museum-tour encoding task in walking AR and stationary VR experimental conditions. The study specifically investigates essential EM aspects, including “What,” “Where,” “When,” and Binding, utilizing recognition and cued recognition measures. To achieve this, thirteen synthetically generated human portraits were used as items to be learned, each rendered in 3D form and associated with a distinct spatial context when presented to participants. Indeed, locomotion was experimentally controlled by using stationary VR and walking AR conditions, whereas encoding events were presented in one of the three different locations categories: 6 presented at mid-route locations, 4 at dead-end locations, and 3 at stairway-adjacent locations.

In a comparison of outcomes between conditions, it was hypothesized that participants in the walking AR condition would outperform their stationary VR counterparts in measures of “What”, “Where”, “When”, and Binding EM aspects. The overall results confirmed a significant positive modulation of EM by locomotion, specifically in “When” and Binding EM aspects. Specifically, participants in the AR condition showed more accurate item, and source order judgements and more accurate item-to-source, as well as source-to-item associations. Additionally, AR participants were able to make more accurate associative inferences from items and sources not seen together at encoding, but linked through common spatial context.

These results are consistent with accounts of episodic specialisation in rapid, one-trial extraction of differences, regularities and cross-modal associations, which support incidental learning of experience^1,18,167–172^. In addition, these findings highlight that spatial learning relies not only on the integration of visual cues from the environment, but also on idiothetic cues derived from locomotion, which in this study were available to participants in the walking AR condition^158,173,174^. Moreover, this interpretation is supported by a match between the objective advantages in “When” and Binding, and, significantly greater confidence self-reports for AR condition participants across IQ and DQ^175,176^.

In a second objective, this study aimed to investigate how EM could be influenced by the EFs encountered during each encoding event at the different stimuli-presentation category locations. To this end, the experiment categorized stimuli-presentation locations in 6 mid-route locations, 4 dead-end locations, and 3 stairway-adjacent locations. Concerning stairway-adjacent stimuli presentation-events, the hypothesis posited that increased attention concentration and event prediction processes upon encountering the stairway would improve encoding and provide context-rich information for recall^47,48^. In stairway negotiation, a twofold effect was expected. First, a change in the cognitive SMC model of the environment, and the construction of a new space model ahead, which would induce an increase of encoding performance for items immediately adjacent to the stairway^31,43,72^. Second, a relational cluster switch would imply differences between pre- and post-stairway relational measures, such as order judgements and associative inferences between items and places^45,49^.

In a comparison of EM measures between stimuli-presentations locations, results showed a significant performance increase effect of stairway-adjacent locations compared to dead-end and mid-route stimuli-presentation locations. Considering the changes between pre- and post-stimulus presentation, while dead-end locations primarily introduced new prediction challenges in the motor aspect of SMC model of the environment, staircase-adjacent locations were the sole stimulus-presentation events which simultaneously challenged the existing predictions, in both the visual and motor aspects of the engagement with the environment.

Additionally, an in-depth examination of how each measure of EM interacted with each location type, showed that performances from stairway-adjacent locations were enhanced in all EM measures (i.e., “What”, “When”, “Where”, Binding), but more noticeably in “Where” (i.e., source recognition) and Binding scores (i.e., item-to-source associations). Moreover, to assess the boundary role of the stairway in the organisation of EM retrieval, accurate responses from participants were divided in pre- and post- staircase presentations. A significant advantage was found in correct serial order recall of pre-stairway human portraits items in comparison to those encountered post-stairway, irrespective of condition.

These results support the hypothesis that the integration of multimodal sensory cues and idiothetic information obtained through locomotion is crucial for constructing cognitive relational models of the spatial context in which events unfold^13,162^. Furthermore, it showed that, similarly to route turns^30^ and doorways^164,166^, stairways are integrated in the relational cognitive model of spatial context. Because of the challenge posited by the stairway, affecting both visual and motor aspects of the predictive engagement with the environment, this EF is incorporated into the relational cognitive model of space as a kind of event boundary^29,31,72^.

Furthermore, results confirmed a differential serial recall accuracy for target items encountered pre- and post- event boundary^49^. The experience of level-shift via the stairway divided the cognitive relational representation of events encoded during the museum-tour into two clusters^45^. As a consequence, there is an increased likelihood of within-cluster items to be recalled in correct order compared to cross-cluster serial order judgements.

This study presented some limitations that should be taken into account. First, given the sample size of the present study, the generalisation of these findings should be taken with caution. Second, while previous research, consistent with the findings presented in this article, has demonstrated that immersion levels in VR and AR setups do not impact EM performance^115–118^ future studies could delve deeper into this subject. Moreover, in this experiment, participants’ decisions regarding the encoding itinerary were controlled under both conditions. Future studies should address this aspect, as previous research has identified volition over the itinerary as a modulating factor for certain aspects of EM^101,102,177^. In this context, it is possible that a greater agency over navigation in an encoding itinerary could lead to different EM outcomes. Additionally, despite participants not reporting the use of mnemonic techniques, a remaining concern is the implementation of strategies to prevent rehearsal during the navigation segments between stimuli exposures. In the current experimental design, this is a possibility that cannot be excluded for participants in the VR condition. In order to extend our knowledge of the mechanisms involved in EM construction, future work could implement specific strategies to prevent rehearsal during navigation, as in^109^. Although this study controlled the auditory and visual aspects of an encoding experience, as well as the idiothetic signals derived from locomotion (e.g., vestibular and proprioceptive), future studies could include other sensory modalities (e.g., odour) to help unravel their role in modulating EM processes^178–182^. Previous research has shown that the availability of olfactory cues at encoding would be integrated in the memory of the episode^183–185^, the pleasantness and arousal of olfactory information and the congruency between encoding and retrieval could interact with specific EM aspects^186–189^.

Despite these limitations, the findings of this study carry both theoretical and practical implications. They highlighted a positive impact of locomotion on episodic memory, particularly in the realms of context recognition and item-source relational measures. Additionally, this research unveils, for the first time, an event-boundary effect associated with stairways in episodic memory, indicating improved performance and a clustering effect in the recall of events before and after encountering the stairway.

We believe the findings of the current study have potential applications in various fields, particularly in enhancing existing and developing new design criteria for neurocognitive rehabilitative interventions targeting pathological aging, EM and spatial processing impairments using VR and AR technologies^114,190–193^. Previous research suggests that locomotion in VR environments can enhance accuracy in spatial learning and object localization tasks^194^, and facilitate cognitive map building^195^ even in the absence of visual information. Moreover, engaging in active navigation within augmented and virtual environments has been identified as advantageous for spatial memory tasks among older adults^196^. Research findings indicate that salient landmarks may help spatial navigation and memory tasks in individuals with AD, aMCI and dementia patients^197,198^. Hence, VR and AR may represent viable alternatives enabling the exposure to different locomotion scenarios and incidental memorisation tasks, tailored for each specific case requirements^191,192,199,200^. Finally, the controlled lifelike encoding task, along with the underlying AR/VR framework developed for this study, provides researchers with an opportunity to conduct controlled yet ecologically valid experiments, allowing for the naturalistic observation of EM processes.

## Methods

Using a between-subjects design, a controlled experiment was carried out at the Caixaforum Museum in Barcelona, Spain from February 5th to May 30th, 2022. The incidental encoding task simulated a tour of the Caixaforum Museum. Pre-trial instructions to participants asked them to rate the faces of potential tour guides, on the basis of personal preferences considering both the face and the Museum location in which each portrait was found. No instructions related to learning, memory or mnemonics were provided before trials. During the encoding tour, thirteen stimuli were presented to participants in AR and VR conditions. Each stimulus consisted of a 3D-rendered photorealistic human portrait at a specific location of the Museum. Both experimental conditions shared the same tour-origin location, as well as each stimulus and its location was held constant across trials. EM performance and organisation were assessed immediately after tour completion and 48-hours after trials using a Remember/Familiar (RF) recognition paradigm. The experiment’s instructions, questionnaires, and debriefing were administered in Spanish.

This study received approval from the Ethical Committee of the Open University of Catalonia (UOC), Spain. All participants gave their informed consent to the study, which was performed in compliance with the Declaration of Helsinki^201^ and UOC’s ethical committee guidelines.

### Participants

#### Sample size

In order to calculate a minimum sample size, we assessed similar past studies and their reported effect sizes, specifically interested on face recognition, locomotion and encoding that occurs while navigating through spatial boundaries. An a priori power analysis for ANOVA repeated measures between subjects was carried out using G*Power 3.1^202,203^. For the estimation, a Cohen’s *F* of 0.4 (large-size effect) was used, together with a level of power of 0.8, and an $\alpha$ level of 0.05. The minimum sample size returned 28.

#### Recruitment and allocation

The recruitment campaign was carried out through online and offline means in the University environment in Barcelona. Individuals were ineligible for the current study if they had been diagnosed with psychiatric disorders, had memory complaints or spatial navigation impairments, individuals using prescribed or recreational drugs, pregnant people and persons unable to walk unattended. The recruitment campaign obtained fifty eight respondents (32 female), from which twenty eight healthy adults (19 female) met this experiment’s inclusion criteria and voluntarily agreed to participate. Participants in this sample were aged between 21 and 59 years old $(M=39.91,\ SD=9.88)$.

Aiming to improve the power of the study by reducing the variability between groups, participants were randomly assigned to experimental conditions following an age-based stratified randomisation process^204–206^. When a subject met the inclusion criteria and agreed to participate, she was first allocated to a corresponding age-based bin, and the corresponding experimental condition was then assigned through randomisation on the age bin. Randomisation was performed using a computer-based true random integer generator, using age bins in ranges $(18 - 24,\ (Mdn=21)$, $(25 - 34 ,\ (Mdn=29.5)$, $(35 - 44 ,\ (Mdn=39.5)$, $(45 - 54 ,\ (Mdn=49.5)$, $(55 - 64,\ (Mdn=59.5)$.

### Materials

A technological framework was specifically developed using Unity3D/C# to implement the site-specific incidental encoding task consistently across VR and AR modalities. The experimental task design provisioned fourteen graphical markers used for the presentation of comparable 3D human portraits at consistent Museum locations in AR and VR conditions. These fourteen markers were tailored for this study to ensure a high degree of distinctiveness from other graphical elements in the Museum. Each graphical marker included a visually distinct symbol providing direction (i.e., an arrow) and distance (i.e., number of steps) to the next location in the museum-tour. These markers were printed on A4 paper and placed in independent floor-standing information displays measuring 1 meter in height. The markers were visible in both experimental conditions. Each stimulus presentation was held consistent across conditions in duration, in size and in visual perspective respective to the environment.

#### Stimuli

The present study employed photorealistic human portraits as target stimuli. This choice was based on past research which has shown that faces are processed faster than other object types^207,208^, and draw more attention than other visual stimuli^209,210^, even in complex social environments^211^ as it was the case in the current experimental design. And specifically, positive emotional signals on faces have been reported to enhance face recognition memory^212^. Moreover, the use of faces as stimuli has increasingly become a focus of cognitive assessment tools and research in AD and aMCI, processes associated with the decline in EM function^213–216^.

**Figure 4:**
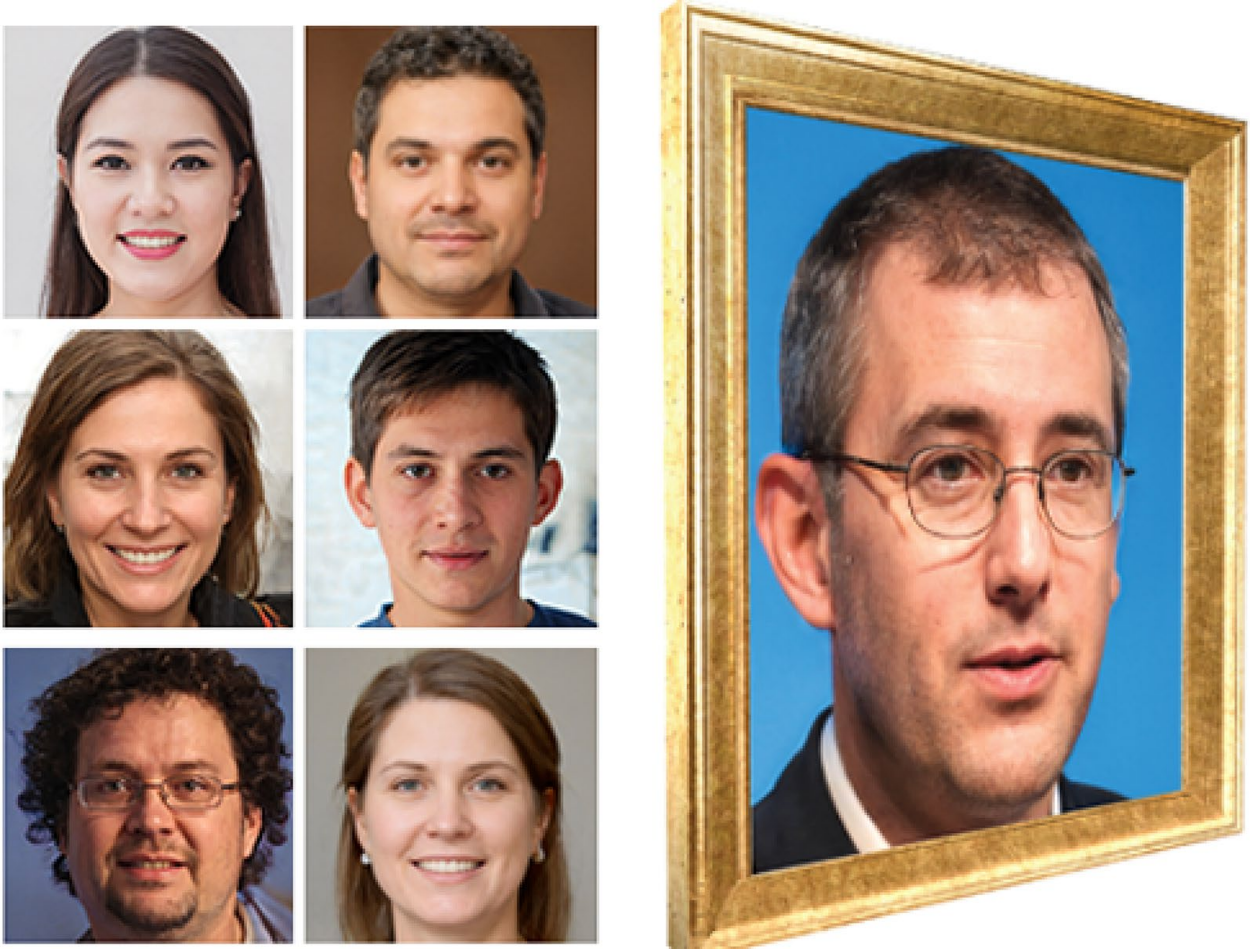
Left. Examples of the photorealistic human face portraits produced using generative adversarial networks to ensure their novelty to participants. Right. Each synthetic portrait to be learned was rendered in 3D form and mounted in a wooden frame to resemble a Museum object.

To ensure that the face stimuli had not been previously seen by participants, photorealistic facial portraits were generated using the styleGAN2 method^217^. A total of 100 synthetic portraits were produced, from which fourteen images were manually selected, with gender, skin colour, background color, and eyeglasses as balancing factors (Figure 4-Left). Each portrait selected was rendered in a wood frame as a 3D object to closely resemble a Museum object (Figure 4-Right). Thirteen 3D objects served as target items, while one was reserved as a pre-trial practice item.

The resulting 3D target items were consistent in terms of visual complexity in features that could influence attention or memory encoding. Previous research has suggested that *beta* and *gamma* parameters of a Weibull distribution can capture clutter, spatial coherence, and complexity of an image, and that these parameters are strongly correlated with neural activity^218^. We used a similar approach to^219^ to estimate beta and gamma parameters of a Weibull distribution from the face portraits utilised as stimuli in the study. A Levene’s Test for homogeneity of variances showed no significant differences between the gamma and beta Weibull values of the stimuli images, grouped per building level for beta $(p=.3)$ and gamma parameters $(p=.1)$. Independent samples t-tests showed no significant difference between the stimuli images when grouped per building level for beta $(t(11) = -1.01, p = .3)$ and for gamma $(t(11) = 0.097, p = .9)$. This indicated that the face stimuli used were similar in visual complexity.

Considering that past studies have suggested that positive emotional signals on faces may enhance face recognition memory^212^ in this study, the Python-based software tool Py-Feat was used to obtain a computational measure for displayed facial emotion signals^220^. This method uses a six emotion model^221^ plus a neutral state. Results of this inferential procedure showed for Happy $(n=13, M=.95 SD =0.085)$ while the next runner-up was detected as Neutral $(n=13 M=0.023 SD=.047)$. A Levene’s Test for homogeneity of variances showed no significant differences between the values of emotional inferences obtained via Py-Feat when grouped per building level for Happy $(p=.1)$ or for neutral $(p=.2)$. A Mann-Whitney U Test showed no significant difference between emotional gesture displayed by the stimuli images when grouped per building level for Happy $(U = 18.0, p =.7 )$ or for Neutral $(U = 19.0, p =.6)$. This analysis highlighted that the synthetic faces used in this experiment overall showed a happy facial gesture, and that the display of emotional signals among stimuli was similar.

The design of the current study controlled that each of the encoding events presented the target stimulus with equal visual characteristics across conditions. Specifically the distance and perspective towards target, lighting conditions and stimulus size. Both in AR and VR, each encoding event displayed the target stimulus for a continuous 9-second interval.

#### Location

The experimental environment included both the ground floor and the first level of the Caixaforum Museum, including a two-way mechanical escalator linking the two levels. The origin of the encoding tour was set on the ground floor of the Museum and was held consistent across trials in both conditions.

**Figure 5:**
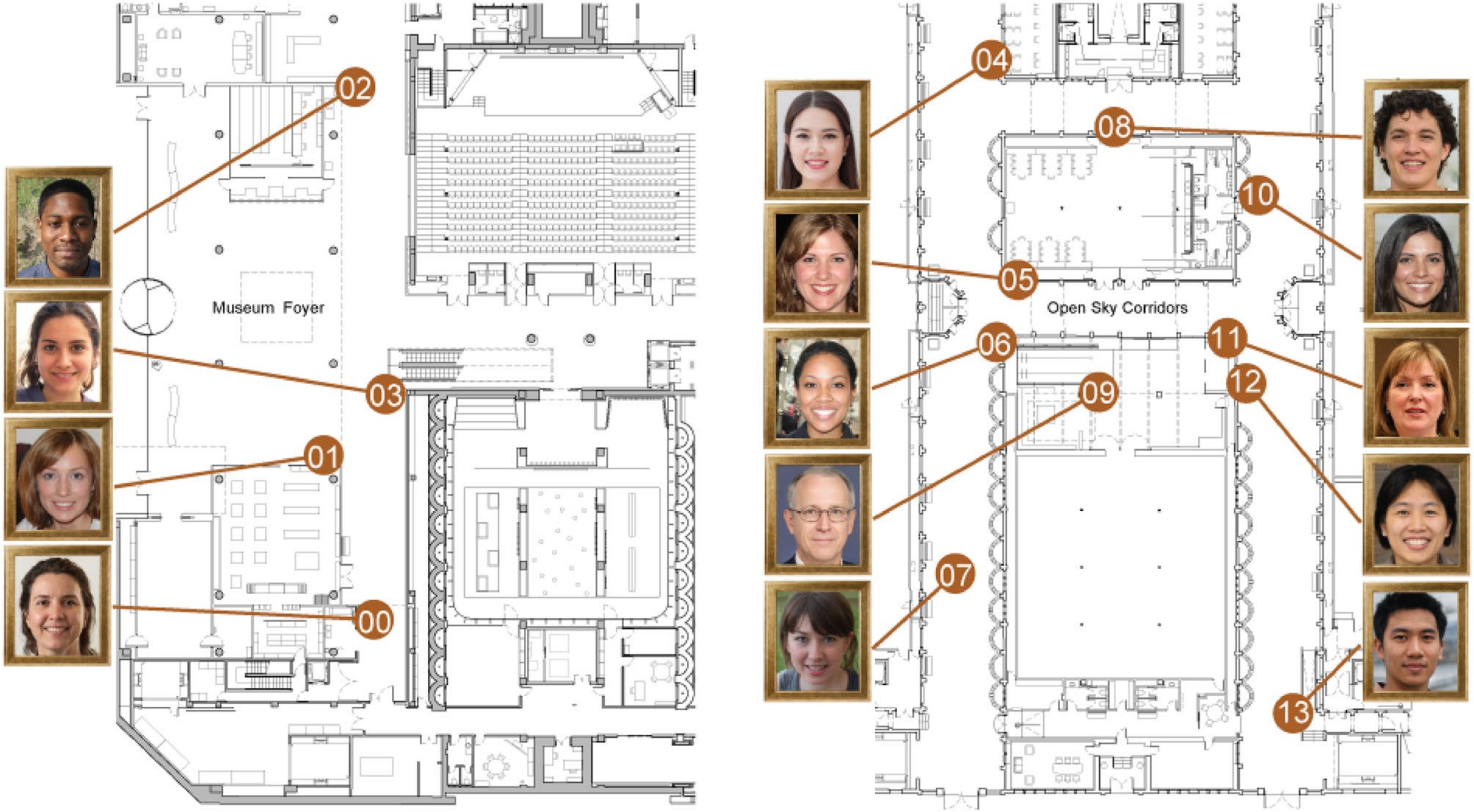
Ground floor (Left) and first floor (Right) plans of the experimental environment. The *museum-tour* encoding task started from a consistent location across trials (location 00, pre-trial practice item). The encoding tour included thirteen stimuli-presentation locations (01-13), distributed on the ground floor and first floor of the Museum.

The thirteen Museum locations were selected as stimulus-presentation locations and target items were positioned at these locations consistently across trials (Figure 5). The museum-tour included only public areas and excluded all access-regulated places (i.e., exhibition areas, restrooms, Museum shop, Museum cafe). The resulting encoding tour was approximately 500 meters in length and took approximately 12 minutes to complete by walking.

#### Stimulus-presentation locations

In this study a characterisation of the available stimulus-presentation locations was performed, in an approach that follows^153^ to how the built environment may contribute to neurodynamics of memory^59,154–156^.

**Figure 6:**
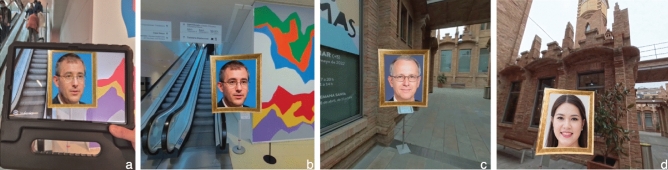
Examples of stimuli presented at specific locations. Each stimulus presentation was held consistent in both the AR condition (**a**) and the VR condition (b,c,d) in duration, in size and in visual perspective respective to the environment. This study used three stimuli-presentation locations: Stairway-adjacent (**a**,**b**), mid-route (**c**), dead-end (**d**).

The stimuli-presentation locations used in this study included 6 mid-route locations (i.e., least visual change, least motor change between pre- and post-stimulus presentation. See Figure 6-c), 4 dead-end locations (i.e., most motor change, but least visual change. See Figure 6-d), 3 stairway-adjacent locations (i.e., most motor change and most visual change. See Figure 6-b).

#### Augmented reality condition

Participants in the AR condition were given specific task instructions at the origin location of the museum-tour. Then, a familiarisation phase was conducted, which included examining the AR-ready handheld device, demonstrating a sample printed graphical marker, and testing the stimulus presentation procedure. During this familiarisation phase, it was highlighted that each graphical marker included a visible arrow pointing in the direction of the next target and an approximate number of steps to reach it. Once the familiarisation phase was completed, the AR tour started from the origin of the museum-tour. Participants walked the museum-tour following the proposed route, and at the encounter of each printed marker participants followed the procedure to acquire the graphical marker using the device’s camera. Each procedure triggered the display of a target 3D portrait for a continuous 9-second interval. Following the exposure, participants were prompted to rate each presentation-event by selecting either “yes” or “no” on the device’s touchscreen in response to the question “Did you like it?”. Participants resumed the museum-tour after submitting a vote. The AR condition was implemented using Unity3D/C# and Vuforia as a standalone application running on a Samsung S7 tablet with custom plastic grips, displaying 2560 by 1600 pixels over a 28 cm by 18 cm screen.

#### Virtual reality condition

In preparation of the experimental campaign, a stereoscopic video of the museum-tour was recorded using a Vuze XR camera. This recording registered the tour at a leisurely pace, with all graphical markers at their corresponding Museum locations. The result was an 13 minutes long video with a resolution of 4096 $\times$ 2160 pixels and a frame rate of 60 frames per second, displaying stereoscopic information within a 180^∘^ horizontal field of view.

During the trials, participants in VR condition sat down on a chair at the origin location of the museum-tour. They received specific task instructions and were equipped with a head-mounted display (HMD) and a hand-held controller to interact with the VR application. Next, they started a familiarisation phase, which included the presentation of a stimulus sample and the corresponding interaction to vote using the hand-held controller. The VR tour started once the familiarisation phase was completed. Once participants started the tour, the stereoscopic video displayed the first segment of navigation towards the first stimulus location. The VR tour paused automatically in front of the first graphical marker to display the associated portrait continuously for 9 seconds. Immediately after a stimulus presentation, participants were prompted to cast their vote on the portrait by answering “yes” or “no” to the question “Did you like it?”. Once participants submitted a vote by pressing a button on the hand-held controller, the tour resumed. Each consecutive segment of the tour, stimulus presentation and voting, were carried out following the same procedure, until returning to the origin location of the tour. The tour was consistent across all trials, and included the presentation of the same set of stimuli at identical locations, preserving the visual perspective in relation to context, stimuli size, and duration.

The VR condition was implemented using Unity3D/C# and resulted in a standalone application. It was administered individually to each participant via an Oculus Quest2 HMD and using one hand-held controller. The HMD visual display used 3840 x 1920 pixels.

### Measures

Post-trial EM measures were collected via an IQ and a DQ using custom Qualtrics questionnaires. The questionnaires collected general demographic information (e.g.,, age, sex, education) as well as information on participants’ familiarity with the Museum building using a 5-point scale. It also inquired about whether any mnemonic technique was used during the tour.

#### Emotional self-report at the start of retrieval

A considerable body research has highlighted how certain emotional states can hinder participants’ retrieval performances. For instance, lure discrimination ability and recognition memory^222,223^, and depression compounded by anxiety^224^, have shown to yield an adverse effect on immediate recall amount of acquisition, and on the retrieval of newly learned information. Also importantly, low trait anxious subjects may be more likely to assign remember judgement to a false memory when they are stressed^225^. In order to detect emotional states, such as anxious, stressed or depressed participants, this study implemented at the start of the IQ and DQ memory measures an abbreviated version of the Discrete Emotion Questionnaire (DEQ)^226,227^. The DEQ measure implemented in this study was based on self-report of emotional state, using a seven-point Likert scale, per each of the components of a six-emotion model (i.e., Anger, Disgust, Surprise, Happiness, Fear, Sadness)^221^.

#### Confidence self-reports

As recommended by previous studies^228,229^, confidence self-reports can help to clarify the contributions of familiarity and recollection^83^. Confidence self-reports in this study were registered from “When” and Binding responses (i.e., direct item/source associations, inferred item/source associations, time duration estimation and serial order judgements). Participants registered a self-report of confidence associative question using a five-point Likert scale from “Totally confident” to “Not confident at all”.

#### What, Where, When, Binding

The core of the EM questionnaires included a RF recognition procedure and a forced-choice (FC) recognition procedure. Both IQ and DQ contained RF face recognition and place recognition sections, FC place recognition and face recognition sections, face and place order judgement sections, and face-based and place-based associative inferences. A tour duration estimation section was only present in the IQ. The questions in IQ and DQ used both, stimuli seen by participants at encoding, and distractors or lures. Lures were image items of faces and places that were not part of the encoding tour, but shared strong similarities with the stimuli presented at encoding.

The IQ and DQ examination of EM aspects “What”, “When”, “Where” and Binding was accomplished using the following sections:

- Face and associated place.
  This section of the questionnaires was twofold: first a RF face recognition task examining the “What” EM component. Followed by an associated place FC task focused on item/source Binding. The face recognition task consisted in the presentation of a specific portrait alongside the question “Did you encounter this person?” and three possible responses: “Yes I Remember”, “Seems Familiar”, “Not Encountered”. If participants selected either the Remember or Familiar response, the FC task asked them to identify the location where they had encountered the portrait by selecting between two images in response to the question “Where did you encounter this person?”. When they answered “Not Encountered”, the questionnaire continued directly to the next question. The face and associated place section was presented in 14 trials in the IQ and 7 trials in the DQ.
  The face lures presented in this section were selected from the same database of synthetic faces generated for this experiment. These portraits were not encountered by participants during the encoding phase but shared strong similarities (i.e., facial characteristics, sex, skin color, glasses, hairstyle, and background color) with the stimuli presented to participants. Similarly, the lure images of places used in the FC task depicted locations from the Museum building (i.e., featuring similar architectural elements) but which were excluded from the experimental tour, thus not seen by participants at encoding.
- Place and associated face.
  The place and associated face recognition section of the questionnaires was implemented using a RF place recognition question focused on the “Where” EM aspect. Followed by an associated face FC question, which examined source/item Binding. In this case, a specific image location was presented alongside the question “Did you encounter this place?” with three possible responses: “Yes I Remember”, “Seems Familiar”, “Not Encountered”. Participants were then asked to choose between two portraits to answer the question “Who did you encounter at this place?”, if they answered either “Yes I Remember” or “Seems Familiar”, otherwise, the questionnaire continued with a new question. The place and associated face recognition section consisted of 12 trials in the IQ and 7 trials in the DQ. Lure images of places and faces were produced using the same procedure as described in the previous section.
- Order judgement of Faces and Locations.
  This section focused on examining “When” EM aspect, requiring participants to recall the temporal sequence of the stimuli encountered during the experimental route. Participants were asked “Which did you encounter first?” and presented with pairs of previously seen places or faces, and asked via FC to identify the correct order of occurrence (i.e., the first item they encountered during the route)^150^. This section was divided equally into face and place order judgments and consisted of 18 trials in the IQ and 8 trials in the DQ. This section contained no lure, nor for locations, nor for faces.
- Time duration estimation
  In addition to the “Order Judgment of Faces and Locations” section of the questionnaire, another question aimed to examine the “When” aspect of EM. Participants were asked to estimate the duration of their museum-tour using a horizontal slider with minute-scale resolution. The slider ranged from 5 to 30 minutes, with the default value set to 5 minutes. This question was only presented in the IQ.
- Associative inferences based on spatial context.
  To further examine Binding, the questionnaire included an associative inferences section, which aimed to measure participants’ ability to form cross-connections between items (faces and locations) that were indirectly linked through common spatial context. Each trial presented a triad of images comprising a base-associator, which was either a face (i.e., face-based) or a place (i.e., place-based), seen by participants at encoding, plus two image options, either faces or places which were also part of the encoding tour, but with different spatial proximity relations to the base-associator. The associative inference was prompted using the question “Which was nearer?” (i.e., which of the two image options was closer to the base-associator). This section was divided equally between face-based and place-based associative inferences and comprised 18 trials in the IQ and 4 trials in the DQ. No lures were included in this section.

### Procedure

Participants were assigned individual time slots to participate in the experiment. To ensure comparable lighting conditions, trials for the AR condition were scheduled at times consistent with the time when the stereoscopic video of the museum-tour was recorded for the VR condition. Upon arrival at the Museum, the research team welcomed participants, explained the experiment’s course and requirements, and solved questions from participants regarding the study. Finally, participants provided written informed consent to take part in this study. After this, participants were taken to the origin location of the experimental tour, situated on the ground floor of the Museum.

In both conditions, the experiment began with a familiarisation and practice stage conducted individually with each participant. Then, they were instructed to perform a museum-tour, in which an undisclosed number of Museum *tour guides* would be encountered. Participants were asked to rate the faces of these tour guides, on the basis of subjective preferences, considering both the face and the Museum location in which each portrait was presented. No instructions related to learning, memory or mnemonics were provided before trials.

Next, specific instructions were given to participants in both conditions regarding how to engage with each stimulus presentation and cast their vote in the corresponding AR or VR graphical interface. Participants were advised that virtual objects would be visible only for a brief period of time, requiring their full attention. For VR participants only, additional instructions asked them to report any nausea or dizziness they experienced immediately after completing their tour.

The museum-tour origin location was the same for both conditions, and all VR environmental conditions were matched to those encountered during the AR condition. This included the location of each stimulus in the Museum, presentation distance to target item, and the size and visual perspective in which each stimulus was presented (Figure 5). The tour finished at the origin location for both conditions.

After completing an encoding museum-tour, each participant, irrespective of condition, was invited to sit alone at a table in front of a computer screen and complete the IQ concerning their tour experience. They were instructed to respond to each question carefully, without time limit, and were advised to seek help if required. The entire procedure took approximately 60 minutes for each participant. After completing the experimental procedure, participants were thanked and received an entrance ticket to the Museum exhibitions.

Forty-eight hours after participating in the experiment, participants received an email inviting them to answer the DQ. DQ completion took approximately 25 minutes for each participant. After completing the 48-hour later task, participants were thanked and debriefed.

### Statistical analyses

Data from participants were collected post-trial via EM questionnaires administered immediately after task completion (IQ) and 48 hours later (DQ). Statistics were performed using JASP computer software^230^. In this study, $\alpha$ values less than 0.05 were considered as statistically significant. Assumptions underlying parametric tests were verified and upheld if not otherwise specified. Where violations to these assumptions occurred, non-parametric equivalent tests were used.

When possible Student’s T-Test or Mann-Witney U Tests were employed however when multiple variables were ANOVA was preferred and when comparisons over time were attempted RMANOVA were used. In ANOVA $\omega ^2$ is reported because it has been acknowledged as a lesser biased alternative to $\eta$ family, especially when sample sizes are small^231–235^. Bonferroni control for Type-I errors were performed in this study. This is in line with previous studies highlighting that Bonferroni is the less tolerant method for false positives^236,237^. Also Bonferroni has been suggested more suitable for small sample sizes^238,239^. Due to this study’s sample size, low tolerance for false positives, and the potential correlation between tests’ results, Bonferroni was deemed the most suitable procedure against Type-I errors^240,241^. To interpret effect sizes and correlations we followed^242^, and the guidelines from^243,244^.

#### Preliminary data analyses

To ensure the comparability between groups of participants and between the encoding experiences of VR and AR conditions, the following analyses were conducted.

First, a post-hoc Levene’s Test for equality of variances and a Mann-Whitney U Test were performed, to verify the homogeneity in the distribution of age of participants between conditions.

Next, to examine the homogeneity between the encoding experiences of participants in AR and VR conditions three comparisons were performed: Missed items in questionnaire sections, total duration of encoding tour, and differences in ISI. A RMANOVA was performed comparing the variances of the number of questionnaire measurements available to each subject between conditions, using each of the seventeen different sections of the IQ and DQ as measures. An ANOVA and Kruskal-Wallis Tests were used to compare the differences in tour duration between conditions. A RMANOVA and Kruskal-Wallis Tests were used to compare the variances in inter stimulus intervals between conditions using the values.

Furthermore, to verify that the level of knowledge of the building (i.e., the experimental site) was comparable between conditions, a Mann-Whitney U Test was performed using scores obtained via linear transformation of 5-point Likert scale responses.

Finally, to assess the emotional state of participants at the beginning of the retrieval measures, a DEQ measure was implemented both at IQ and DQ. DEQ was based on self-report of emotional state using a seven-point Likert scale, per each of the components of a six-emotion model (i.e., Anger, Disgust, Surprise, Happiness, Fear, Sadness)^221^. To assess each of these components of emotional state in a comparison between conditions, an ANOVA was conducted using scores obtained via linear transformation from Likert scale ratings^245^.

#### Analyses for H1

This study focused first its analyses on the What, Where, When and Binding aspects of EM. To examine the What and Where components the analysis focused on accurate responses from RF face recognition responses and place recognition responses at IQ and DQ. To examine the When component the analysis focused on the accurate responses in a FC serial order task on IQ and DQ, and a tour-duration estimation task at IQ. Binding was examined via FC considering both face-to-place and place-to-face correct associations, as well as face- and place-based correct associative inferences. To make these assessments Student’s T-tests and Mann-Whitney U Tests were employed. When significant differences of the separate IQ and DQ measures were found, further examination of responses over time was performed using RMANOVA. Post-hoc Bonferroni corrections were employed in the report of these results.

Next, an overall average score was calculated for each EM aspect “What”, “Where”, “When” and Binding using combined IQ and DQ responses. To further characterise these results, average scores were computed for all EM aspects “What”, “Where”, “When” and Binding, separately for IQ and DQ. To finalise this characterisation of the results, an overall all-aspect score was computed (OER) per subject, averaging accuracy scores obtained at IQ and DQ from all EM measures of “What”, “Where”, “When” and Binding. In this case, Mann-Whitney U Tests were used to examine differences between conditions.

#### Analyses for H2

In the second group of analyses, this study was focused on the potential influence on EM of the EFs encountered at encoding. In this analyses, stimulus-presentation locations were grouped in three types: mid-route (6 locations), dead-end (4 locations) , and stairway-adjacent (3 locations) . Mid-route locations were characterised as imposing the least amount of visual change, least motor change between pre- and post-stimulus presentation. Dead-end, were characterised as imposing the most amount of motor change, but least of visual change. And stairway-adjacent were characterised as imposing the most amount of motor change and of visual change together.

Responses from face recognition (“What”), place recognition (“Where”), item-to-source association, source-to-item association (direct Binding), as well as face- and place-based associative inferences (inferred Binding), were considered compounded, as an all-aspect EM average for an ANOVA between the three location types. To further characterise this exploration, responses from face recognition (“What”), place recognition (“Where”), item-to-source association, source-to-item association (direct Binding), as well as face- and place-based associative inferences (inferred Binding), were considered separately as “What”, “Where”, “direct Binding” and “inferred Binding” score types and used in an ANOVA comparing between three location types and four score types. In these analyses Standard Post-hoc tests, Kruskal-Wallis Tests, Dunn’s Tests and Bonferroni corrections were employed.

The last analysis examined if the stairway had an effect on serial-order responses, which was a measure of “When” EM component. To this end, face and place serial order responses were divided in pre-stairway and post-stairway stimuli presentation-events and used in Mann-Whitney U Tests.

#### Confidence Self-reports

Confidence self-reports were obtained using a seven-point Likert scale in the questionnaire items of IQ and DQ that addressed “When” and Binding segments. For comparisons between conditions, Student’s T-tests and Mann-Whitney U Tests were conducted, using scores obtained via linear transformation from Likert scale ratings^245^.

### Supplementary Information

Supplementary Information.

## Data Availability

Collected and analysed data during the current study are available from the corresponding author upon reasonable request.
