# Supplementary material for: Comparing episodic memory outcomes from walking augmented reality and stationary virtual reality encoding experiences
Source: Sci Rep. 2024 Mar 30;14:7580. doi: 10.1038/s41598-024-57668-w (PMC10981735; doi:10.1038/s41598-024-57668-w)
Supplement: Supplementary file 1 — Supplementary Information. [file 41598_2024_57668_MOESM1_ESM.pdf]

Synthetic view of all the tests conducted in this study.

Aspect	Score	Test	Compared	Results of interest
What	Face recognition IQ	Mann Whitney U test	WAR, SVR	
	Face recognition DQ	Mann Whitney U test	WAR, SVR	
	Face recognition IQ and DQ	RMANOVA	WAR, SVR	
Where	Place recognition IQ	Mann Whitney U test	WAR, SVR	
	Place recognition DQ	Mann Whitney U test	WAR, SVR	
	Place recognition IQ and DQ	RMANOVA	WAR, SVR	
When	Duration estimation	Mann Whitney U test	WAR, SVR	
	Face order judgement IQ	Mann Whitney U test	WAR, SVR	p=.02
	Place order judgement IQ	Mann Whitney U test	WAR, SVR	
	Face order judgement DQ	Mann Whitney U test	WAR, SVR	p=.02
	Place order judgement DQ	Mann Whitney U test	WAR, SVR	
	Face order IQ and DQ	RMANOVA	WAR, SVR	p=.01
	Place order IQ and DQ	RMANOVA	WAR, SVR	
Binding	Associated face IQ	Student's T-test	WAR, SVR	
	Associated place IQ	Mann Whitney U test	WAR, SVR	
	Associated face DQ	Mann Whitney U test	WAR, SVR	p=.01
	Associated place DQ	Mann Whitney U test	WAR, SVR	p=.004
	Face based associative inference IQ	Student's T test	WAR, SVR	
	Place based associative inference IQ	Student's T test	WAR, SVR	
	Face based associative inference DQ	Mann Whitney U test	WAR, SVR	p=.005
	Place based associative inference DQ	Mann Whitney U test	WAR, SVR	
	Associated face IQ and DQ	RMANOVA	WAR, SVR	
	Associated place IQ and DQ	RMANOVA	WAR, SVR	p=-02
	Face based associative inference IQ and DQ	RMANOVA	WAR, SVR	p=.002
	Place based associative inference IQ and DQ	RMANOVA	WAR, SVR	p=.04
Calculated	What IQ	Mann Whitney U test	WAR, SVR	
	WHAT DQ	Mann Whitney U test	WAR, SVR	
	What IQ and DQ	Mann Whitney U test	WAR, SVR	
	WHERE IQ	Mann Whitney U test	WAR, SVR	
	WHERE DQ	Mann Whitney U test	WAR, SVR	
	Where IQ and DQ	Mann Whitney U test	WAR, SVR	
	WHEN IQ	Mann Whitney U test	WAR, SVR	
	WHEN DQ	Mann Whitney U test	WAR, SVR	
	When IQ and DQ	Mann Whitney U test	WAR, SVR	
	BINDING IQ	Mann Whitney U test	WAR, SVR	p=.04
	BINDING DQ	Mann Whitney U test	WAR, SVR	p<.001
	Binding IQ and DQ	Mann Whitney U test	WAR, SVR	p=.003
	All EM aspect IQ	Mann Whitney U test	WAR, SVR	
	All EM Aspect DQ	Mann-Whitney U Test	WAR, SVR	p=.003
	OER	Mann-Whitney U Test	WAR, SVR	p=.04
EFs	Place Order Scores	Mann Whitney U Test	WAR, SVR	
	Face Order Scores	Mann Whitney U Test	PreS, PostS	p=.046
	What, Where, Binding	ANOVA, Kruskal Wallis, Standard Post-Hoc test, Bonferroni	DE,SA,MR	MR pBonf =.006 and DE pBonf=.012 both in favour of SA
	What, Where, Binding compound score	ANOVA, Kruskal Wallis, Dunn's	DE,SA,MR	MR Pbonf=.04 and DE p<.001 both in favour of SA

Forty-four (44) tests run in total. Used abbreviations: Immediate questionnaire (IQ); 48-hour later questionnaire (DQ); analysis of variance (ANOVA); repeated measures ANOVA (RMANOVA); Overall Episodic Recall (OER); Walking AR (WAR); Stationary VR (SVR); PreS (Pre-stairway); PostS (Post-stairway); Dead-end (DE);Stairway-adjacent (SA); Mid-route(MR).
